# Psychological impact of autologous hematopoietic stem cell transplantation in systemic sclerosis patients and influence of resilience

**DOI:** 10.3389/fimmu.2024.1436639

**Published:** 2024-10-24

**Authors:** Marc Schmalzing, Michael Gernert, Matthias Fröhlich, Jörg Henes, Nathalie Schwindl, Leona Zerhusen, Lukas Berthold, Johannes Hewig, Andrea Kübler, Ann-Christin Pecher, Sonja Kleih-Dahms, Patrick-Pascal Strunz, Philipp Ziebell

**Affiliations:** ^1^ Department of Internal Medicine 2, Rheumatology/Clinical Immunology, University Hospital Würzburg, Würzburg, Germany; ^2^ Centre for Interdisciplinary Clinical Immunology, Rheumatology and Auto-inflammatory Diseases and Department of Internal Medicine II (Oncology, Hematology, Immunology, Rheumatology), University Hospital Tübingen, Tübingen, Germany; ^3^ Institute of Psychology, University of Würzburg, Würzburg, Germany

**Keywords:** systemic sclerosis, psychological impact, autologous hematopoietic stem cell transplantation, resilience, immunosupression

## Abstract

**Objective:**

In severe cases of systemic sclerosis (SSc), autologous hematopoietic stem cell transplantation (aHSCT) is superior compared to cyclophosphamide. But treatment related morbidity and mortality have to be considered. To date, data on major physical and psychological impacts of aHSCT are scarce. Therefore, subjectively experienced physical and psychological impact of aHSCT and exploration of internal and external factors helping to cope with aHSCT was assessed.

**Methods:**

Retrospective assessment of physical and psychological variables in an SSc cohort after aHSCT to describe: Health-related quality of life (HRQL), SSc-associated impairment, coping strategies, body image, and resilience. Additionally, semi-structured interviews were conducted and analyzed via mixed methods qualitative content analysis.

**Results:**

Thirty-two patients were included. HRQL correlated with impairment due to SSc and with depressive coping. An unfavorable body image correlated with reduced HRQL and increased impairment but improves after aHSCT. Patients with good resilience had a better HRQL, less depressive coping, and less SSc-associated impairment. The semi-structured interviews revealed that resilience is important for a successful disease management as patients with higher resilience were more satisfied with aHSCT, patients with lower resilience would have wished for more psychological support. Thirty-one patients would recommend aHSCT to other patients.

**Conclusion:**

A transient negative impact of aHSCT on mental well-being is present but can be relieved by a team specialized to aHSCT. Psychological support seems to be an unmet need, particularly in patients with low resilience. Patients with higher resilience described a lower negative impact caused by aHSCT and higher satisfaction after therapy.

## Introduction

Systemic sclerosis (SSc) is characterized by vasculopathy, inflammation, and fibrosis (1). Therefore, different pharmacological treatments are needed to address the different components of SSc. Progression of lung fibrosis can be slowed by nintedanib (2), vasculopathy, especially pulmonary arterial hypertension, can be improved by phosphodiesterase 5 inhibitors, guanylate cyclase stimulators, endothelin receptor antagonists, prostacyclin analogues, and prostacyclin receptor agonists (3), and inflammation can be treated by methotrexate (4), mycophenolate (5), tocilizumab (6), rituximab (7), and cyclophosphamide (CYC) (8). As SSc has the highest case mortality of all rheumatic diseases, mainly due to pulmonary hypertension and lung fibrosis (9), a stronger immunosuppression is needed if the aforementioned therapies are not efficacious. In this situation, autologous hematopoietic stem cell transplantation (aHSCT) is the treatment modality with the best evidence to date. Three randomized controlled trials [i.e. ASSIST (10), ASTIS (11), and SCOT (12)] showed superiority of aHSCT versus CYC concerning lung involvement, skin involvement and overall survival. Hence, treatment-related morbidity and mortality have to be considered, before performing such an intensive treatment. Not only somatic factors can influence the outcome after aHSCT, but also psychological factors play an important role in the management of aHSCT and are important for a successful course of aHSCT.

It has to be considered that many SSc patients experience a low health related quality of life, due to impairment caused by the sclerosis (13). Severity and disfigurement of aggressive disease forms have a major impact on psychological well-being (14). Additionally, many SSc patients are not satisfied with their body image (15). All of this results in a high prevalence of depression among SSc patients (16). An intense treatment such as aHSCT can have an additional negative impact on mental well-being. For example, in hematological malignancies, several studies demonstrated self-reported depression and reduced quality of life during and after hospitalization for aHSCT (17). Factors that influence the psychological processing of the experiences during aHSCT are coping strategies, resilience, and internal control belief. Validated questionnaires exist to assess these parameters. Coping comprises strategies to handle stress and it is modifiable depending on situation and personality (18), whereas resilience describes psychological strength in the light of challenging life situations and is thought to be a stable trait throughout an individual’s life (19). High resilience is protective of psychiatric disorders in the face of trauma or stress. Control beliefs can be external or internal. Patients with external control beliefs are convinced that they cannot control their surroundings and that their health is under control of other people. Patients with internal control beliefs think that their health mainly depends on their own efforts. In this way, control beliefs influence health related issues differently (20). To date, only one study is published describing the psychological impact of aHSCT in patients with diffuse cutaneous SSc (21), using semi-structured interviews. The qualitative analysis gave valuable insights that the patients would not have expected such an intensity of treatment and that they would have wished for more psychological support, but a quantitative psychological assessment of patients or a correlation of psychological parameters with certain interview statements was not done.

The aim of this study was to assess and correlate physical and psychological well-being in SSc patients after aHSCT at two German expert centers, and to identify patients at risk for a strong impact of aHSCT on psychological well-being and quality of life by using a quantitative assessment of psychological parameters comprising body image, coping strategies, control beliefs, and resilience. Based on the aforementioned literature, we hypothesized for the first part of our study:

- Health related quality of life (HRQL) correlates with inversely with subjective impairment due to SSc.
- HRQL correlates inversely with depressive coping.
- Unfavorable body image correlates with reduced HRQL and increased subjective impairment.
- Unfavorable body image improves after aHSCT.
- Patients with good resilience have better HRQL.
- Patients with good resilience have less depressive coping.
- Patients with good resilience have less SSc-associated impairment.

Moreover, in the second part of our study additional explorative and qualitative analysis was performed, comprising structured telephone interviews, and thereby this study also investigated if patients with unfavorable psychological evaluation express more need for psychological support.

## Patients and methods

### Patients

Thirty-two patients were included. All patients fulfilled the ACR/EULAR classification criteria of 2013 for SSc (22) and had gone through aHSCT analogous to the ASTIS trial between the years 2008 to 2020 at the University Hospital of Tübingen or the University Hospital of Würzburg. Patients were contacted by telephone or during their regular follow-up visit at the outpatient department of these centers and were asked for enrolment into the study. Patient acquisition took place from 2021 to 2022. Only adult patients were included who did not decline a telephone interview.

### Quantitative and qualitative data collection

Patients were assessed in a two-step approach: In the first step, quantitative questionnaires were used to cover selected physical and psychological variables. To assess physical and mental health-related quality of life the 36-item short form health survey (SF-36) (23) was used, which includes two subscales the physical component score (PCS) and the mental component score (MCS). To assess the amount of impairment due to SSc the scleroderma health assessment questionnaire (SHAQ) (24) was used. Body image was assessed with the adapted satisfaction with appearance scale (ASWAP) (25), and coping strategies with the *Freiburger Fragebogen zur Krankheitsverarbeitung* (FKV-15) (26). The FKV-15 is based on the theoretical foundation of Lazarus and Folkman’s (18, 27) transactional stress model, and includes scales on coping styles such as *active coping* (adaptive and problem-focused), *distraction* (gaining distance), *trivialization* (downplaying), *compliance* (trusting the medical professionals and following their instructions), and *depressive coping* (maladaptive and emotion-focused). Each of the scales can fluctuate between 1 and 5, with 5 being interpreted as a strong expression of this coping style. Resilience was assessed with the *Resilienzskala* (RS-11) (28). The questionnaires were sent to the patients by mail. Only when patients returned all completed questionnaires, they were contacted by one of three interviewers by telephone for the second step of this study, for qualitative semi-structured interviews. Interviewers were two students of psychology and one medical student, who were trained and supervised by two rheumatologists and one psychologist. Patients were informed beforehand that their questionnaires and interview contents would be blinded to their treating physicians to prevent social-desirability bias. Semi-structured interviews based on Spierings et al. (21) and Bresser et al. (29) included personal expectations for and experiences during treatment, coping, support by family or professionals, recovery experience, suggestions for improvement. The interviews were divided into four parts according to Misoch et al. (30). Patients were asked open-end questions to allow unexpected answers and obtain topics most important to the patients.

### Quantitative and qualitative analysis

For the quantitative analysis of the questionnaires, means and standard deviations were calculated. Cronbach’s alpha was calculated to test the validity of the questionnaires. One-sided Pearson’s correlations and t-tests were calculated to test our hypotheses (alpha-error-level conventionally *p* = 0.05). Microsoft Excel (Redmond, Washington) and SPSS (IBM, Armonk, New York) were used as statistic programs. For the interviews, transcription was performed with easy-transcript (https://www.e-werkzeug.eu/index.php/de/produkte/easytranscript), followed by a mixed methods qualitative content analysis with an explorative inductive category development according to Mayring (31, 32), utilizing MAXQDA Analytics Pro 2018 (https://www.maxqda.de/produkte/maxqda-analytics-pro).

## Results

### Study population and questionnaires

Thirty-two SSc patients were included, 17 female, 15 male, with a mean age of 51.5 years (range 29 – 69 years). The SSc specific characteristics are shown in **Table 1**. The mean time between aHSCT and telephone interviews was 5.8 years (standard deviation [SD] ± 3.8). All questionnaires, except for body image, refer to the time when this study was conducted. Most of the questionnaires exhibited an acceptable to excellent internal consistency. An overview of the results of all questionnaires is shown in **Table 2**. Several outcome parameters could have influenced the satisfaction with the aHSCT and the answers in the questionnaires. Therefore, patients with elevated CRP, with a low improvement of the mRSS of less than 10 points after aHSCT, and patients with SSc relapse after aHSCT were analyzed but did not have differences compared to patients without this poor prognostic parameters in the questionnaires. Nine patients needed immunosuppression after aHSCT, mostly due to progress auf SSc. These nine patients received 15 immunosuppressive drugs, i.e. mostly more than one drug per patient. **Table 1** shows the drugs initiated after aHSCT.

**Table 1:** Characteristics of the study population before and after aHSCT.

Characteristics	Values
Female, *n* (%)	17/32 (53.1)
Age, mean (range), years	51.5 (29-69)
Time between study and aHSCT, mean (SD), years	5.8 (3.8)
CRP elevation (> ULN) before aHSCT, *n* (%)	9/32 (28.1)
mRSS before aHSCT, mean (SD), points	21.3 (11.0)
mRSS after aHSCT, mean (SD), points	6.5 (5.0)
Lung involvement, *n* (%)	27/32 (84.4)
Cardiac involvement^§^, *n* (%)	15/32 (46.9)
Renal involvement, *n* (%)	1/32 (3.1)
Anti-Scl-70 antibody positivity, *n* (%)	26/32 (81.3)
Immunosuppressive medication before aHSCT, *n* (%)	
Cyclophosphamide	24/32 (75.0)
Mycophenolate	12/32 (37.5)
Methotrexate	13/32 (40.6)
Rituximab	5/32 (15.6)
Hydroxychloroquine	3/32 (9.4)
Ciclosporin A	2/32 (6.3)
Azathioprine	2/32 (6.3)
Sulfasalazine	1/32 (3.1)
Patients needing immunosuppressive medication after aHSCT, *n* (%)	9/32 (28.1)
Cyclophosphamide	2/32 (6.3)
Mycophenolate	2/32(6.3)
Methotrexate	3/32 (9.4)
Rituximab	6/32 (18.8)
Azathioprine	2/32 (6.3)
Time point of immunosuppression after aHSCT, median (IQR), months	12.0 (8.5)

*aHSCT* autologous hematopoietic stem cell transplantation, *IQR* interquartile range, *mRSS* modified Rodnan skin score, *SD* standard deviation, *ULN* upper limit of normal.

^§^i.e. high-sensitive troponin above upper limit of normal + myocardial late enhancement in cardiac MRI or myocarditis in myocardial biopsy.

**Table 2:** Results of all questionnaires.

Questionaire	Subscale	Mean	Standard deviation	Range	Cronbach’s α
SF-36	Physical component	58.36	23.67	0-100	0.78
	Mental component	66.15	22.03	0-100	0.83
SHAQ		0.88	0.56	0-3	0.90
FKV-15	Depressive coping	2.27	0.80	1-5	0.82
RS-11		60.31	10.88	11-77	0.89
ASWAP	Body image before aHSCT	36.66	17.22	0-84	0.61
	Body image after aHSCT	28.09	15.86	0-84	0.69

*AHSCT* autologous hematopoietic stem cell transplantation, *ASWAP* adapted satisfaction with appearance scale, *FKV-15* Freiburger Fragebogen zur Krankheitsverarbeitung, *RS-11* resilience scale, *SF-36* 36-item short form health survey, *SHAQ* scleroderma health assessment questionnaire.

### Health related quality of life correlates with subjective impairment and depression

**Figure 1:**
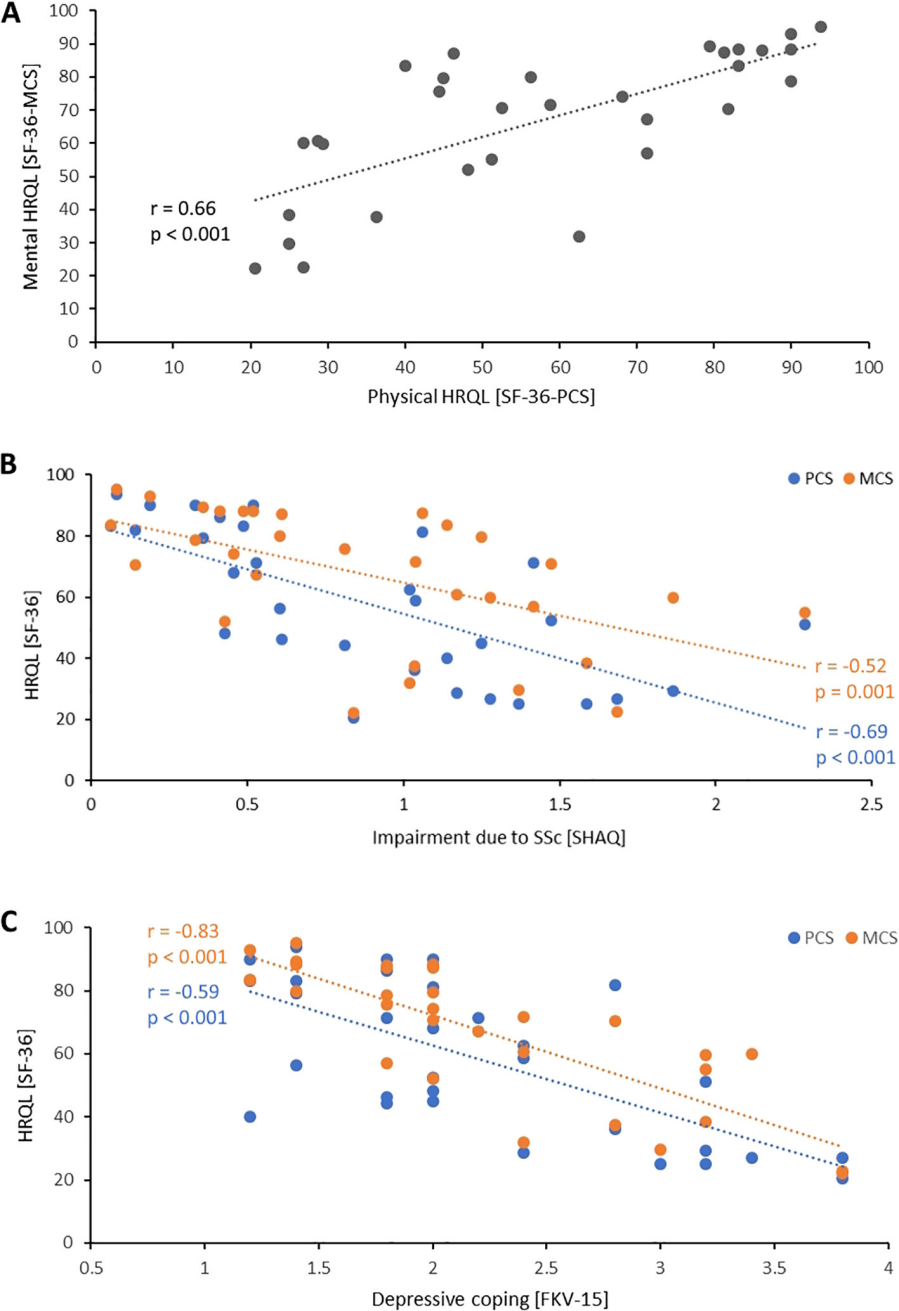
Interrelationship of health-related quality of life with impairment and depressive coping. **(A)** Correlation between physical and mental health-related quality of life. **(B)** Correlation between physical health-related quality of life (blue) or mental health-related quality of life (orange) with subjective impairment due to SSc assessed with SHAQ. **(C)** Correlation between physical (blue) and mental health-related quality of life (orange) with depressive coping. Each dot indicates one patient. FKV-15, *Fragebogen zur Krankheitsverarbeitung*; HRQL, Health-related quality of life; MCS, Mental component scale; PMS, Physical component scale; SF-36, 36-item short form health survey; SHAQ, Scleroderma health assessment questionnaire.

HRQL was assessed with the SF-36. A significant correlation between the physical component score (PCS) and the mental component score (MCS) was seen (*r* = 0.66, *p* < 0.001) **Figure 1A**. A high subjective impairment due to SSc correlated with low physical and mental quality of life (SF-36-PCS ~ SHAQ: *r* = -0.69, *p* < 0.001; SF-36-MCS ~ SHAQ: *r* = -0.52, *p* = 0.001) **Figure 1B**. Physical and mental HRQL correlated inversely with depressive coping (SF-36-PCS ~ FKV-15: *r* = -0.59, *p* < 0.001; SF-36-MCS ~ FKV-15: *r* = -0.83, *p* < 0.001) **Figure 1C**.

### Unfavorable body image correlates with reduced HRQL and increased impairment but improves after aHSCT

**Figure 2:**
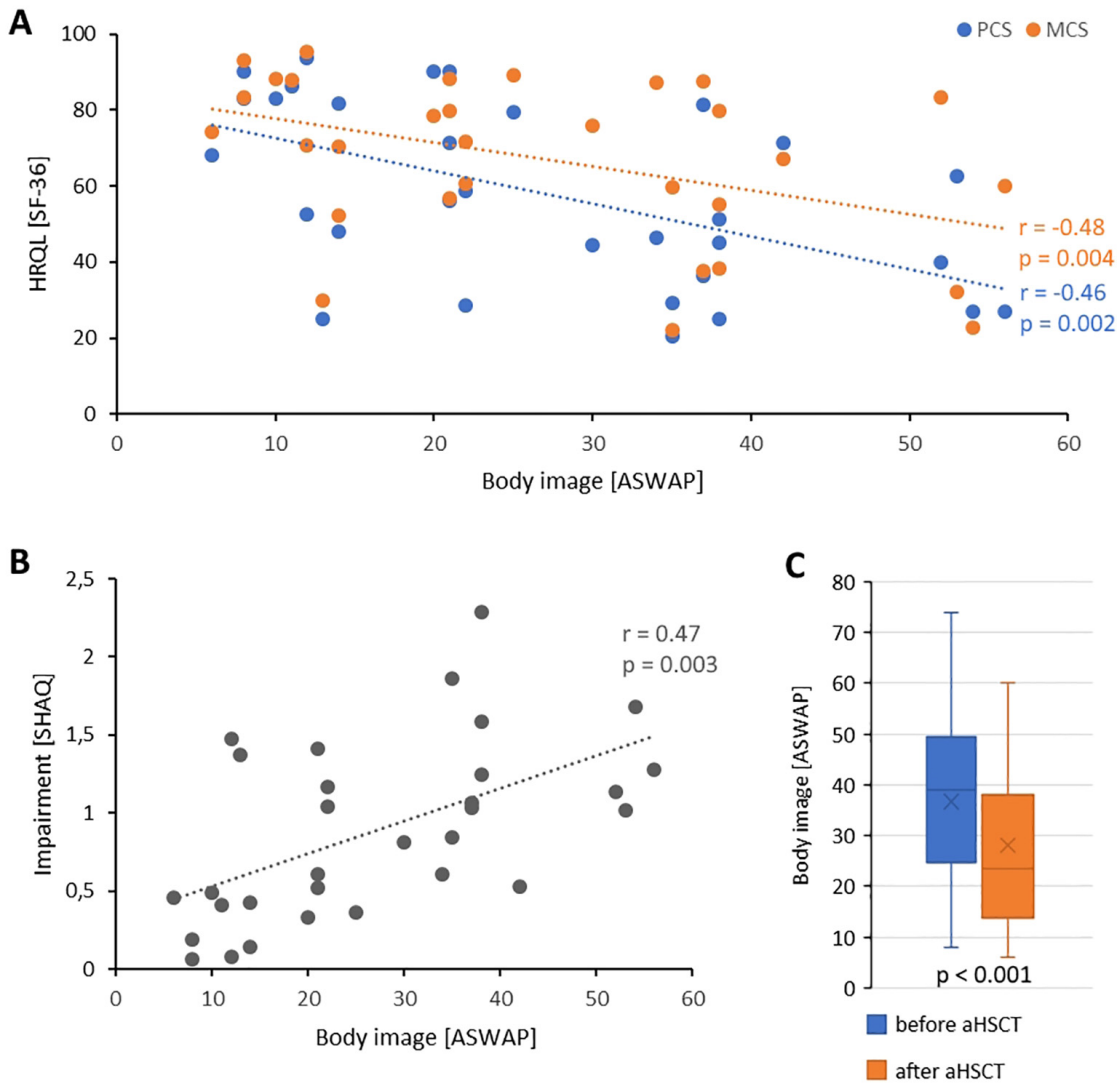
Body image in the setting of aHSCT. **(A)** Correlation between physical health-related quality of life (blue) or mental health-related quality of life (orange) with body image. **(B)** Correlation of body image with subjective impairment due to SSc. Each dot indicates one patient. **(C)** Body image before aHSCT and after aHSCT. Boxplots show medians with 25^th^ and 75^th^ percentiles, whiskers show minimums or maximums. X is mean. ASWAP, adapted satisfaction with appearance scale; HRQL, Health-related quality of life; MCS, Mental component scale; PMS, Physical component scale; SF-36, 36-item short form health survey; SHAQ, Scleroderma health assessment questionnaire.

The body image was assessed with ASWAP, whereas higher values indicate more problems with the acceptance of appearance. An unfavorable body image correlated with a reduced health-related quality of life (ASWAP ~ SF-36-PCS: *r* = -0.48, *p* = 0.004; ASWAP ~ SF-36-MCS: *r* = -0.46, *p* = 0.002) and with a higher subjective impairment due to SSc (ASWAP ~ SHAQ: *r* = 0.47, *p* = 0.003) **Figures 2A, B**.

The body image was retrospectively assessed for the time before and after aHSCT. The ASWAP before aHSCT was 36.7 (± 17.2) and after aHSCT it was 28.0 (± 15.9; *p* < 0.001) **Figure 2C**.

### Patients with good resilience have better HRQL, less depressive coping, and less SSc-associated impairment

**Figure 3:**
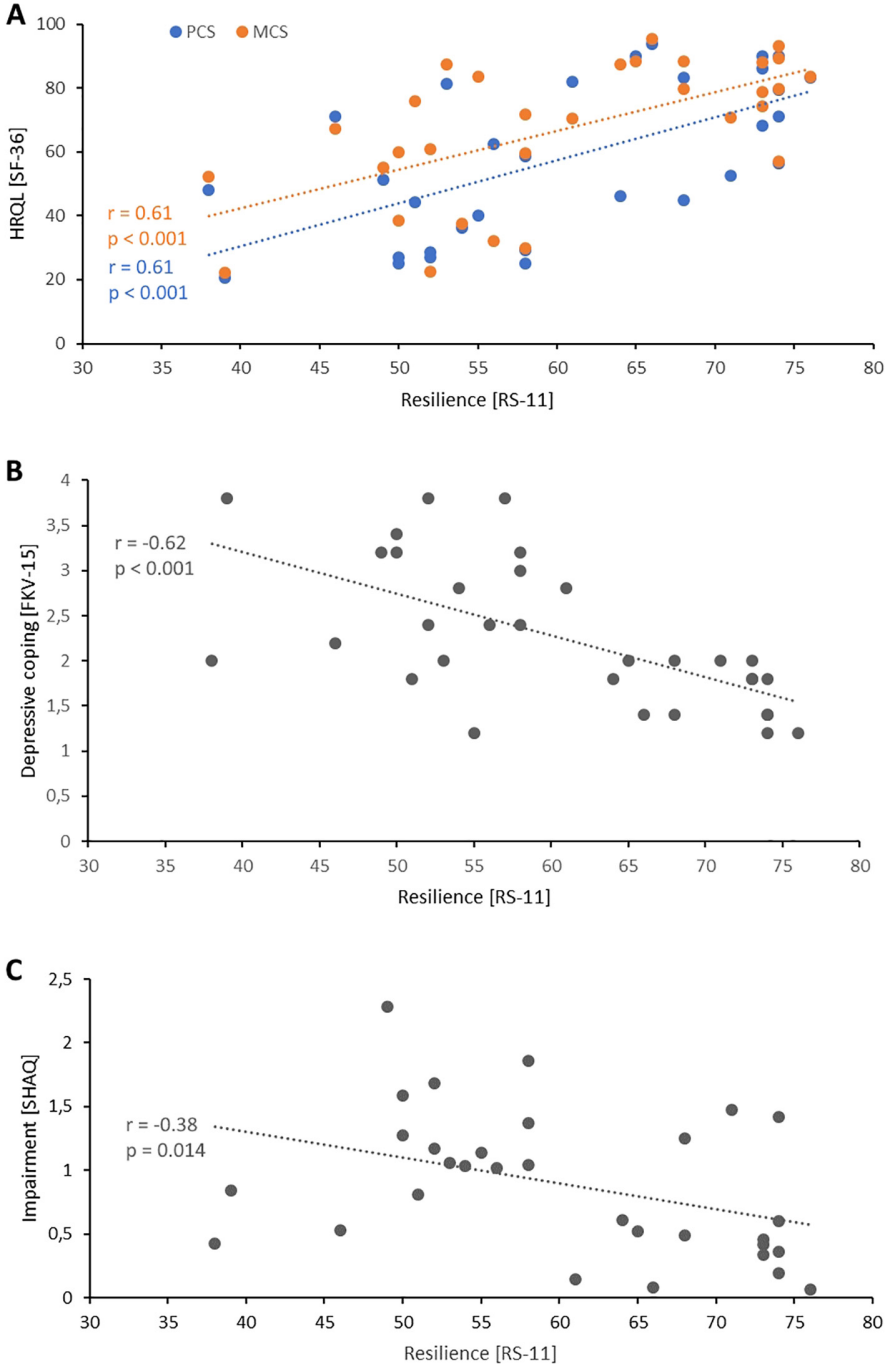
Resilience in the context of SSc. **(A)** Correlation between physical quality of life (blue) or mental quality of life (orange) with resilience. **(B)** Correlation between resilience and depressive coping. **(C)** Correlation between resilience and impairment due to SSc. Each dot indicates one patient. FKV-15, *Freiburger Fragebogen zur Krankheitsverarbeitung*; HRQL, Health-related quality of life; MCS, Mental component scale; PMS, Physical component scale; RS-11, *Resilienzskala*; SF-36, 36-item short form health survey; SHAQ, Scleroderma health assessment questionnaire.

Patients with higher resilience, assessed by RS-11, exhibited a higher physical and mental HRQL (*r* = 0.61, *p* < 0.001 and *r* = 0.61, *p* < 0.001, respectively) **Figure 3A**. In concordance, patients with higher resilience had less depressive coping (*r* = -0.62, *p* < 0.001) **Figure 3B**. Furthermore, a weak inverse correlation was found between resilience and SSc-associated impairment (*r* = -0.38, *p* = 0.014) **Figure 3C**.

### Main findings in the semi-structured interviews

The mean duration of the semi-structured interviews was 59:52 minutes (range 28:37 minutes – 2:29:17 h) per patient. The answers of the patients were collected and grouped as shown in **Table 3**.

**Table 3:** Structure of the patients’ answers form interviews.

Category	Subcategory	Most common answers	Numbers
Information about treatment	Source of information	Physician	30
		Internet	12
		Other SSc patients	9
	Reasons for treatment	AHSCT as last treatment option	28
		Worries about prognosis	17
		Entrustment in the physician	16
	Subjective preparedness for treatment	Good prepared	26
		Bad prepared	1
	Emphasized information	Risk of aHSCT	16
		Course of therapy	14
Expectations	Positive expectations	Positive influence on SSc	25
		AHSCT will stop SSc	13
		AHSCT will cure SSc	4
	Negative expectations	No fears	18
		Fears	14
		Fear of death	10
	Feelings before aHSCT	Wait and see	14
		Over challenged	6
		Positive actionism	5
Experiences before aHSCT	SSc symptoms	Skin thickening	26
		Circulatory disorders	17
		Respiratory problems	15
		Pain	11
	Social surrounding	Support by family	14
		Social distancing	14
		No change in contacts	13
	Functional impairments	Professional restrictions	24
		Mobility impairments	13
	Appearance	Burden	15
		No problems	12
		Feels unattractive	7
Experiences during aHSCT	Feelings	Loneliness	10
		Loss of control	9
		Stable emotions	9
	Management of treatment	Optimism	10
		Diversion	9
	Recommendation of aHSCT to others	Yes	31
	Medical support	Satisfied with clinic	25
		Support through nurses	20
		Support through physicians	19
	Social support	Family	25
		Partner	15
		Friends	9
	Psychological support	No need	16
		Wish for supporting conversations	8
Experiences after aHSCT	Subjective recovery	Positive	24
		Relief	9
		Disappointment	4
	Need for help	Sufficient	15
		Rehabilitation	8
		Psychological help	6
	Course of recovery	Continuous improvements	27
		Powerless	17
		Setback	8
	Employment	Disability pension	12
		Working	9
	Physical changes	Improvement of skin	20
		Persistence of respiratory problems	10
		Persistence of circulatory disorders	10
		Worsening of skin	7
		Fatigue	7
	General condition	Happiness about hospital discharge	15
		Shock about early discharge	4
		Improved quality of life	4
		Feeling of being left alone	4
		Anger about disease	4

The four most important findings were 1) that the majority of the patients would recommend the treatment with aHSCT, 2) many patients were psychologically stressed during therapy, 3) a quarter of the patients would have wished for psychological support during aHSCT, and 4) most of the patients were satisfied with the time after aHSCT, with some limitations.

### The majority of the patients would recommend aHSCT

Thirty-one of 32 patients would recommend an aHSCT for severely affected SSc patients and several patients stated that they had already done so, for example in social media or in self-help groups. Asked if they would recommend the treatment to other patients one patient stated: “Yes, I would definitely do that. Especially, for young people who still have their whole life ahead of them with the disease. [ … ] From an aesthetic point of view, I had expected more. Mouth and hands. But now, I can do everything [ … ]. Who knows what it would have been like if I hadn’t had this stem cell therapy? And maybe I wouldn’t be able to ride a bike anymore. So, I would definitely recommend that to anyone who dares to do it, who is aware that the hair will fall out, who doesn’t worry so much and who would take the risk, I would recommend that immediately.” Reasons and motivations for the SSc patients to accept aHSCT as treatment were the impairment due to SSc and the frustrating experiences with former therapies, as shown in the exemplary quotations: “I wanted to stay alive and worried about my family, especially the kids”, “I had no other choice, because the medication I had received so far did not work” or “I couldn’t even brush my teeth with my right hand, I couldn’t hold a knife or a fork.”

### Many patients are psychologically stressed during aHSCT

Despite their recommendation for aHSCT, 14 patients noted that they had fears, and 10 patients were afraid to die because of therapy (“My fear was not to survive”). Others were less afraid (“After all of the information I gathered, the fear was gone.”). Nine patients felt a loss of control during aHSCT and needed help from the medical staff (“I couldn’t eat, I couldn’t drink [ … ]. I spat, every time. [ … ] you have to go to the bathroom all the time, you can’t walk, so you get a potty.”). Furthermore, being on ward for several weeks with few visits from friends or family was often stressing. Many patients lived far away from the transplantation centers Tübingen or Würzburg, so relatives could not always be there to support the patients. Nevertheless, most of the patients felt support by their surroundings, however, some of the relatives did also struggle with coping: “My husband was quite brisk with me and said, he can’t live with a cripple anymore.”

### A quarter of the patients would have wished for psychological support during aHSCT

Patients needed and cherished the support by the medical staff (“The way the doctors got involved and fought with you, I thought that was great.”). But the additional support by a psychology team, which was not standard care, was deemed necessary by 25% (8/32) of the patients during aHSCT.

### Most of the patients were satisfied with the time after aHSCT

The time after aHSCT was experienced by the majority of patients as satisfying or positive or even better than expected (24 of 32) and 27 patients reported a continuous improvement (“I enjoy my life, I am happy, I am satisfied”, “Compared to before, I am absolutely satisfied because I can live an independent life again, which is worth living for me.”). Expectations to the therapy were realistic. Most of the patients did not expect a major improvement of SSc but expected a stop of illness (“Healing is not possible; I expected a stop of the illness.”).

Nevertheless, four patients were disappointed after aHSCT, for example because a progress of SSc occurred or immunosuppressive treatment had to be restarted (“I would have wished for even more effect. So, the expectations were not quite met, especially because I then needed another drug”). Eight patients reported a setback due to aHSCT. Six patients sought for psychological help after aHSCT (these patients were not identical with the patients, who wished for psychological help during aHSCT).

The emotional impact of aHSCT can persist for long indicated by the fact that our study reactivated bad emotions in some patients (“I probably repressed a lot of things. And when you take the time to fill out this questionnaire, a lot of things came up again that I wasn’t feeling so well on that day.”). The bond between the patient and the physician may persist long after therapy and might have a positive influence on the psychological well-being: “Even today, I still have the name very consciously in my mind. I saw him once during a follow-up examination [ … ]. And he turns around when I was sitting in the waiting area and addresses me by my name. After more than a year, although he was on a completely different ward, and my heart sank because we had such a bond, and he took care of me so touchingly. And when you then experience that a doctor remembers you by name after a year that has touched me very very much.”

### Resilience and non-depressive coping are main characteristics for good disease management

As resilience is assumed to be a stable parameter in personality and less influenced by a retrospective assessment, the patients with bad experiences due to aHSCT were analyzed concerning resilience. Patients, who stated to have felt a loss of control during aHSCT (*n* = 9) had a lower resilience compared to patients, who did not feel a loss of control (RS-11: 54.2 ± 9.6 vs 62.7 ± 10.6). The four patients, who were disappointed with the outcome of the aHSCT had a lower resilience (49.8 ± 7.3) than the not disappointed patients (61.8 ± 10.5; *n* = 28). Patients who wished for psychological support during and after aHSCT had a lower resilience (51.3 ± 9.7; *n* = 8), than patients with high resilience (63.3 ± 9.6; *n* = 24). The latter saw less need for psychological support.

Interestingly, the same patients with low resilience, who felt a loss of control during aHSCT (*n* = 9) and wished for psychological help during aHSCT (*n* = 8) showed a higher depressive coping. The FKV-15 in the patients with felt loss of control was 2.9 ± 0.8 (vs 2.0 ± 0.7 in those who did not report a loss of control) and in the patients, who wished for psychological support the FKV-15 was 2.8 ± 0.8 (vs 2.1 ± 0.7 in those who did not wish for psychological support).

## Discussion

This study retrospectively describes the psychological well-being of SSc patients, who have undergone aHSCT. Most patients were satisfied with the outcome and would recommend aHSCT to other patients but described the impressions during the treatment as intense. Resilience was identified as a character trait that protects patients during and after aHSCT.

The aHSCT aims to improve the somatic components of SSc and is a treatment option for severely affected SSc patients when other immunosuppressive treatments have failed. A systematic review showed that aHSCT in SSc patients is associated with improved physical health-related quality (33). Regarding the psychological component of SSc, improvements in mental quality of life improved only in the ASISST study and did not improve in the ASTIS and SCOT trials.

With the assessed questionnaires a correlation between HRQL and the experienced impairment due to SSc was found. The quality of life decreased with growing impairment. This finding is in concordance with former findings (34, 35). Most important for a reduced HRQL were gastrointestinal involvement, Raynaud’s phenomenon, digital ulcers, dyspnea, and pain (36, 37), as well as a limitation in daily activities, especially limited manual skills (38, 39).

Since SSc-associated problems with appearance often have more impact on the patients’ well-being than pain or dyspnea (40), we chose the ASWAP questionnaire to shine more light on this observation. The ASWAP has been established as a reliable and valid tool to assess the body image in a study with an English SSc sample (25). Our patients describe a worse body image before aHSCT (ASWAP 36.7) compared to a SSc cohort from the USA (ASWAP 32.6) (41). But after aHSCT the body image improved (ASWAP 28.1). Our study hopefully rises the awareness of assessing and improving the body image aspects for SSc patients.

Semi-quantitative interviews assessing the psychological well-being of SSc patients have been performed previously. These interviews by Bresser et al. (29) and Spierings et al. (21) inspired our study. With our interviews we could describe that the most important source of information regarding the aHSCT is the treating rheumatologist. An important motive for undergoing aHSCT was that the patients felt there was no alternative treatment option. Before aHSCT the patients described to be emotionally overstrained, some reacted by withdrawing socially. The negative impact of the disease on social and professional life was confirmed and has already been described in other SSc cohorts (39, 42). During aHSCT many patients experienced a loss of control. The absence of the family was an often-stated burden. Regular visits by the treating rheumatologist were important for the patients and improved their mental well-being. An emotional bond between patient and practitioner often evolved and persisted. After aHSCT most of the patients were satisfied with the course of their convalescence but considered the physical limitations at the beginning of recovery to be challenging. Some patients described the residual symptoms as distressing, but for the majority, acceptance and adaptation prevailed. However, some patients continued to feel burdened and indicated the need for outpatient support.

Resilience is supposed to be a stable trait in a person’s character. Therefore, assessing resilience is hardly influenced by a retrospective study design. The correlation between HRQL and resilience was shown in our SSc cohort and had been described before in other rheumatic diseases (43) and in hematologic patients after autologous and allogenic hematopoietic stem cell transplantation (44). Like in our SSc cohort, patients with hematologic malignancies undergoing stem cell transplantation were more often depressive when they had low resilience (45). Our patients exhibited differences in their resilience, but resilience is supposed to be an important factor for successful coping after stem cell transplantation (46). Our patients with lower resilience were more often unsatisfied with the outcome of the aHSCT and wished for professional psychological help during aHSCT. Therefore, it seems reasonable to assess resilience of each patient before aHSCT to identify persons with low resilience and to offer proactively professional psychological help. Our patients, who wished for psychological support, had a mean value of resilience of 51.3 assessed with the RS-11. This means, that they had a numerically lower resilience compared to the mean resilience of 58.0 from population representative individuals (*n* = 2004) (28).

The major limiting factor of our study is the retrospective character. This implies that patients who have died in the consequence of aHSCT could not be interviewed. Thus, the most negative experiences may not have been recorded. Additionally, the time gap between aHSCT and our study may have weakened the negative experiences. Although we recruited 32 patients for our study, the sample size remains small, which limits its informative value. The ASWAP questionnaire has to date been predominantly investigated in English but was used in a German translation. While we adapted this German version with great care, we acknowledge that it currently comes with certain limitations as reflected in its Cronbach’s α values. Similar to a recent paper on a Swedish version (47), a study that focuses entirely on the translation of the ASWAP would be promising to provide a more reliable and valid German version of this tool for future work. Additionally, the results may have been influenced by the fact that patients responded in a socially desirable manner. In future studies, the psychological well-being should be analyzed also in a cohort of SSc patients that have not undergone aHSCT, to describe the psychological impairments in a generalized SSc group properly.

We want to thank our patients for participating in this study and conclude that an impaired health related quality of life is present in many SSc patients, which should be addressed regularly, especially an unfavorable body image impairs the psychological well-being. During aHSCT a temporary reduction in mental well-being appears, therefore, we suggest to assess resilience before aHSCT to identify patients that may benefit from professional psychological support on ward and afterwards.

## Data Availability

The datasets presented in this article are not readily available due to reasons of sensitivity and are available from the corresponding author upon reasonable request after getting permission from the local ethics committee. Requests to access the datasets should be directed to schmalzing_m@ukw.de.
